# Prof. Theobald Smith

**Published:** 1895-11

**Authors:** 


### PROF. THEOBALD SMITH.

Harvard Veterinarv Department is to be congratulated on the accession to its corps of teachers of the valued services of Prof. Theobald Smith, formerly of the Bureau of Animal Industry, and this must surely prove a strong drawing-card, particularly to those who prefer the field of sanitary veterinary science.

